# Tissue-Based MRI Intensity Standardization: Application to Multicentric Datasets

**DOI:** 10.1155/2012/347120

**Published:** 2012-05-03

**Authors:** Nicolas Robitaille, Abderazzak Mouiha, Burt Crépeault, Fernando Valdivia, Simon Duchesne, The Alzheimer's Disease Neuroimaging Initiative

**Affiliations:** ^1^Centre de Recherche de l'Institut Universitaire en Santé Mentale de Québec, 2601 Chemin de la Canardière, Québec, QC, Canada G1J 2G3; ^2^Radiology Department, Faculty of Medicine, Laval University, Pavillon Ferdinand-Vandry, 1050 Avenue de la Médicine, Québec, QC, Canada G1V 0A6; ^3^Alzheimer's Disease Neuroimaging Initiative, 4150 Clement Street, Building 13 (114M), San Francisco, CA 94121, USA

## Abstract

Intensity standardization in MRI aims at correcting scanner-dependent intensity variations. Existing simple and robust techniques aim at matching the input image histogram onto a standard, while we think that standardization should aim at matching spatially corresponding tissue intensities. In this study, we present a novel automatic technique, called STI for *STandardization of Intensities*, which not only shares the simplicity and robustness of histogram-matching techniques, but also incorporates tissue spatial intensity information. STI uses joint intensity histograms to determine intensity correspondence in each tissue between the input and standard images. We compared STI to an existing histogram-matching technique on two multicentric datasets, Pilot E-ADNI and ADNI, by measuring the intensity error with respect to the standard image after performing nonlinear registration. The Pilot E-ADNI dataset consisted in 3 subjects each scanned in 7 different sites. The ADNI dataset consisted in 795 subjects scanned in more than 50 different sites. STI was superior to the histogram-matching technique, showing significantly better intensity matching for the brain white matter with respect to the standard image.

## 1. Introduction

Magnetic resonance images (MRIs) acquired with similar protocols but on different scanners will show dissimilar intensity values for the same tissue types [1]. These variations are machine-dependent and do not correspond to noise or bias field inhomogeneity, which both can be reduced with different image processing techniques (e.g., [2, 3], resp.). This problem becomes particularly severe in large, multicentric settings such as the Alzheimer's Disease Neuroimaging Initiative (ADNI), in which longitudinal data is being acquired on more than 50 different platforms in the United States and Canada.

Image processing pipelines aimed at extracting tissue-based characteristics (e.g., grey matter/white matter identification) must be robust to these variations. Intensity standardization is therefore employed to reduce interscanner differences in order for similar intensities to have similar tissue meaning in the standardized images, regardless of provenance. It has been shown that standardization improves segmentation [4, 5] and registration [6, 7]. However, scaling intensities with a simple linear transformation is not sufficient, since the influence of the MRI acquisition parameters on the image intensities is nonlinear [6]; a higher order transformation is thus needed.

Published standardization techniques generally propose matching image histograms. An algorithm proposed by Wang et al. [8] stretches or compresses a windowed part of the input image histogram with a multiplicative factor, found by minimizing the bin-count difference between the input and standard images histograms. The window is used to include only pixels of interest and remove, for example, the background; this makes the technique linear in the intensity range of interest. The technique developed by Nyúl et al. [1, 9] matches input image histogram landmarks onto standard histogram landmarks, obtained during a learning process, linearly interpolating intensities between the landmarks. In particular, the variant in [1] uses percentile landmarks, which is simple and more robust. This landmark technique has been used in many studies [5, 7, 10–12]. Jäger et al. [13] extended this principle to two or more jointly used MRI sequences (e.g., T1 and T2), matching multidimensional joint histograms with nonlinear registration. As long as the MRI sequences are spatially aligned, which is assumed, no prior registration of the images is required for computing the joint histogram.

Other techniques use models with some form of *a priori *knowledge, such as the technique proposed by Hellier [6]. It approximates the input image histogram with a mixture of Gaussian functions and aligns their mean with those of the standard image through a polynomial function. Christensen [14] has proposed even-ordered derivatives to find the histogram peak corresponding to the characteristic value of brain white matter; the value is then used to normalize the global image intensity. Weisenfeld and Warfield [4] have proposed modeling the input image as a standardized image corrupted by a linear transformation. Their iterative algorithm then found the parameters of a linear model minimizing the Kullback-Leibler divergence between the standardized and the standard images, thus matching their histograms.

Bergeest and Jäger [15] compared four techniques' performances [1, 4, 6, 13] along with an earlier histogram-matching technique using dynamic histogram warping [16]. None clearly outperformed the others.

Further, in our view, histogram matching should not be the unique objective, as it does not guarantee the standardization of spatially corresponding *tissue* intensities. Towards this end, Leung et al. [17] have recently proposed a semiautomated segmentation technique that delineates cerebrospinal fluid (CSF), white matter (WM) and grey matter (GM) tissue components, for which they computed mean intensities. In a following step, they performed a linear regression between mean intensities and used the results of this regression to perform the standardization. However, this technique yields a linear transformation, which does not completely addresses the problem as mentioned above.

Thus, to our knowledge, techniques presented so far either matched histograms disregarding spatial correspondence or employed spatial correspondence and linear transformations. Our objective was to design a technique that would (1) use both histogram and tissue-specific intensity information; (2) provide a nonlinear intensity transformation between images; (3) share the simplicity and robustness of the Nyúl's landmark technique [1], while remaining fully automated.

In this study, we report the development of our *STandardization of Intensities *(STI) technique, which fulfills these requirements. We compare STI to the variant *L*_4_ of Nyúl et al. [1], which matches foreground (FRG) intensity histograms using decile (10%) landmarks, in two different multicentric T1-weighted MRI datasets: the Pilot European ADNI (Pilot E-ADNI) study and the larger ADNI dataset.

## 2. Methodology

### 2.1. Pilot E-ADNI Dataset

The Pilot E-ADNI dataset was obtained with permission from the multicentric project [18]. Part of this dataset included data from three healthy volunteers, herein referred to as Subjects 1 to 3, scanned within the span of few weeks at seven different European centers (Sites 1 to 7), using the ADNI study 3D T1-weighted MP-RAGE protocol [19]. Information regarding the Pilot E-ADNI study can be found in [18]. Subjects were scanned two times in each center but some data from the first scan were not available. We thus used the data from the second scan only, giving a total of 21 images.

This dataset allowed us to evaluate the performance of standardization techniques by avoiding intersubject intensity variations and focusing only on interscanner differences. Making the reasonable hypothesis that subject tissue properties did not change between sites within the short study timeframe, a well-performing standardization technique should output similar tissue intensities independently of the scanning site.

### 2.2. ADNI Dataset

The second dataset was obtained from the Alzheimer's Disease Neuroimaging Initiative (ADNI) database (http://adni.loni.ucla.edu/). It consisted in 795 baseline MRIs from control, mild cognitive impairment and probable Alzheimer's disease subjects, acquired on more than 50 different 1.5 T scanners (GE Healthcare, Philips Medical Systems, Siemens Medical Solutions) using the aforementioned protocol [19]. Ethics approval was obtained for each institution involved. MR parameters were standardized as per instructions provided by the ADNI MRI Core [19].

The ADNI was launched in 2003 by the National Institute on Aging (NIA), the National Institute of Biomedical Imaging and Bioengineering (NIBIB), the Food and Drug Administration (FDA), private pharmaceutical companies and non-profit organizations, as a $60 million, 5-year public- private partnership. The primary goal of ADNI has been to test whether serial magnetic resonance imaging (MRI), positron emission tomography (PET), other biological markers, and clinical and neuropsychological assessment can be combined to measure the progression of mild cognitive impairment (MCI) and early Alzheimer's disease (AD). Determination of sensitive and specific markers of very early AD progression is intended to aid researchers and clinicians to develop new treatments and monitor their effectiveness, as well as lessen the time and cost of clinical trials.

The Principal Investigator of this initiative is Michael W. Weiner, MD, VA Medical Center and University of California – San Francisco. ADNI is the result of efforts of many co-investigators from a broad range of academic institutions and private corporations, and subjects have been recruited from over 50 sites across the U.S. and Canada. The initial goal of ADNI was to recruit 800 adults, ages 55 to 90, to participate in the research, approximately 200 cognitively normal older individuals to be followed for 3 years, 400 people with MCI to be followed for 3 years and 200 people with early AD to be followed for 2 years. For up-to-date information, see http://www.adni-info.org/.

### 2.3. Standard Image

The standard image used throughout this study is the reference image for the BrainWeb simulation software (http://mouldy.bic.mni.mcgill.ca/brainweb/) [20]. The standard image is a high resolution (1-mm^3^ isotropic), high signal-to-noise average of 27 T1-weighted images of a single human brain, with manually delineated CSF, GM, and WM tissue masks.

### 2.4. Preprocessing

All MRI volumes were preprocessed identically with the MINC image processing toolbox (http://www.bic.mni.mcgill.ca/ServicesSoftware/MINC) before standardization:

1. nonlocal means noise removal [2];
2. intensity inhomogeneity correction [3], performed prior to standardization as suggested in [10];
3. global linear registration (12 degrees of freedom) to the standard image [21], maximizing mutual information between the two volumes [22];
4. resampling to a 1-mm^3^ isotropic grid;
5. intensity clamping, which consisted in (a) setting to zero all intensity values below the percentile value 0.01, (b) setting to 100 all intensity values above the percentile value 99.99, and (c) linearly interpolating intensities between those limits. This step removed outliers of low and high intensities and rescaled image intensity between 0 and 100;
6. global nonlinear registration to the standard image [23], maximizing mutual information between the two volumes as in step 3.

**Figure 1:**
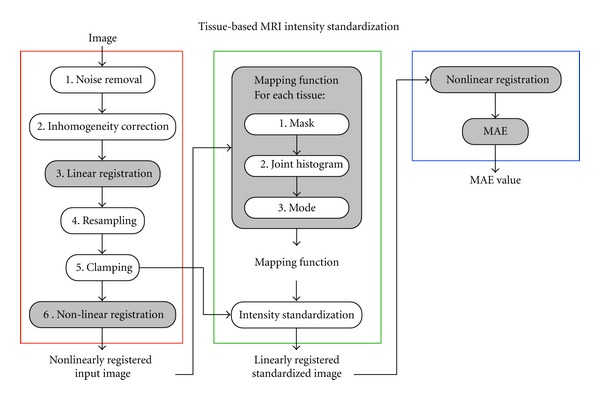
Flowchart showing the steps involved in image preprocessing (red), intensity standardization (green) and mean absolute error (MAE) computation for technique comparison (blue). The steps for which the standard image or its masks are required are shaded in grey. For the *mapping function* process, we summarize the three steps involved in STI.

Left of Figure 1 (red) summarizes the above preprocessing steps.

### 2.5. Intensity Standardization

#### 2.5.1. STI

In the last preprocessing step, global *nonlinear *registration established spatial correspondence between tissues in the standard and input images. This spatial correspondence allowed us to compute a joint intensity histogram of the frequency distribution of intensity correspondences. From the most frequent tissue-specific correspondences, our aim was to compute an intensity transfer function mapping the *nonlinearly registered *input image (preprocessed with steps 1 to 6) onto the standard, which was then applied to the *linearly registered *input image (preprocessed with steps 1 to 5), as shown in Figure 1 (green), giving us the desired standardized image, in the standard reference space for further processing and/or comparison.

Since tissue intensities generally overlap, it was difficult to estimate tissue-specific correspondences from the global joint histogram. To refine its estimates, STI used the standard image tissue masks for (1) background (BKG), (2) WM and (3) GM, from the standard image. We chose to treat the background for two reasons. First, each image can be taken as a whole, with no background removal. Second, intensity corresponding to CSF is often treated in our downstream processing pipelines. Since it is mostly similar to BKG, we found that it was more robust to correct it through BKG standardization.

For each tissue, STI performed the following steps:

1. kept only voxels contained in the applicable tissue mask for both nonlinearly registered input and standard images;
2. computed and smoothed, with a Gaussian low-pass filter, the standard-versus-input joint intensity histogram for the masked voxels. The width of the histogram bins was 0.25% in each dimension, that is, 400 bins covering the 0 to 100 intensity scale, and the full width at half maximum of the Gaussian filter was set to 10 bins;
3. found the mode, that is, maximum, in the joint histogram. The mode corresponds to the most frequent intensity correspondence between the nonlinearly registered input and standard images for the current tissue. This point determined a histogram landmark pair corresponding to the input-to-standard intensity mapping for the current tissue.

To the set of landmark pairs obtained with the tissue masks, STI added two extra pairs: the first, (0, 0), mapped both minimum intensities in the nonlinearly registered input and standard images, and the second, (100, 100), their maximum values. The resulting landmark set *S*_STI_ is then given by

(1) $$\begin{array}{cc} S_{\text{STI}} & =\{\left(0,0\right)\left(m_{r,\text{BKG}},m_{s,\text{BKG}}\right)\left(m_{r,\text{GM}},m_{s,\text{GM}}\right)\\ & \;\;\left(m_{r,\text{WM}},m_{s,\text{WM}}\right)\left(100,100\right)\}, \end{array}$$

where *m*_*r*,*X*_ and *m*_*s*,*X*_ represent the intensity of the nonlinearly registered input and standard images, respectively, for tissue *X*.

STI completed the mapping function by linearly interpolating intensities between the landmark pairs (*piecewise *linear transformation) and finally applied this function to the *linearly* registered input image (preprocessed with steps 1 to 5) in order to create the standardized image.

We added an experimentally determined heuristic to this algorithm. Given large overlaps between tissue classes in some cases, we ordered the search from largest to smallest tissue component, reducing the voxel search space once a component mode was estimated. Practically, this resulted in estimating BKG first, as it generally had the largest mode. Once found, all voxels in an intensity range up to 10% above that mode were removed before estimating the WM mode. After the WM mode had been found, voxels in an intensity range 25% below as well as all voxels above this mode were removed before estimating the GM mode, thereby removing overlap between BKG/GM and GM/WM.

#### 2.5.2. *L*_4_

We compared STI to the following implementation of the histogram-matching technique described in [1] as *L*_4_, which uses decile, that is, 10%, landmarks to match the histograms of nonlinearly registered input and standard images foreground (FRG). FRG is determined in each image via intensity thresholding. It corresponds to the set of voxels for which the intensity is (1) higher than or equal to the mean intensity computed over the whole image and (2) lower than the intensity corresponding to the percentile value 99.8 obtained for the whole image. This operation thus crops the histogram of each image by removing, as assumed, the background and the intensity outliers, respectively. While the clamping step in the preprocessing phase already deals with outliers by clamping the image intensities between percentile values 0.01 and 99.99 to produce the input image, we chose to keep the additional less-tolerant limit of 99.8 to conform with [1].

For both the nonlinearly registered input and standard images, the intensity values corresponding to the percentile value 99.8 were first found, creating a first landmark pair. Decile landmarks, corresponding to the percentile values {10,20,…, 90}, were then determined, within FRG only. This operation yielded nine more landmark pairs.

Two extra pairs, (0, 0) and (100, 100), were finally added to map, respectively, the minimum and the maximum values of the nonlinearly registered input and standard images. We thus obtained the following set of landmark pairs *S*_*L*4_:

(2) $$\begin{array}{cc} S_{L4} & =\{\left(0,0\right)\left(m_{r,\text{fg},10},m_{s,\text{fg},10}\right)\left(m_{r,\text{fg},20},m_{s,\text{fg},20}\right)\\ & \;\;\cdots \left(m_{r,\text{fg},90},m_{s,\text{fg},90}\right)\left(m_{r,99.8},m_{s,99.8}\right)\left(100,100\right)\}, \end{array}$$

where *m*_*r*,fg,*P*_ and *m*_*s*,fg,*P*_ represent the intensities at percentile *P* for the nonlinearly registered input and standard image FRG, and *m*_*r*,99.8_ and *m*_*s*,99.8_ are intensity values at 99.8 percentile for the whole images. The mapping function is obtained by interpolating linearly between landmark pairs.

### 2.6. Technique Comparison

As mentioned, the intensity standardization mapping functions were *determined* using the preprocessed nonlinearly registered images (preprocessing steps 1 to 6). However, the obtained mapping functions were *applied* to the linearly registered images, preprocessed with steps 1 to 5, that is, prior to nonlinear registration.

To compare standardization techniques, we applied, as shown in Figure 1 (blue), a nonlinear registration to the latter images to match the standard image, using the same technique as in preprocessing step 6. We then used the standard image tissue masks to compute the voxelwise mean absolute error (MAE) on different voxel sets: (1) the standard image FRG, as defined in the *L*_4_ procedure above, (2) WM and (3) GM. Applying standardization to the images prior to nonlinear registration allowed us, in this comparison scheme, to reduce any bias toward STI associated with the use of the standard masks.

The MAE was given by

(3) $$\text{MAE}=\frac{1}{N}\overset{N}{\underset{v=1}{\sum }}\left|I_{\text{o},v}-I_{\text{s},v}\right|,$$

where *N* is the number of voxels, and *I*_o,*v*_ and *I*_s,*v*_ are intensity values for the nonlinearly registered standardized output and standard images, respectively, at voxel *v*. We note that since output and standard images intensity scales range from 0 to 100, MAE can be expressed in percentage.

We performed *t*-tests to evaluate the statistical significance of MAE differences between *L*_4_ and STI.

## 3. Results

### 3.1. Pilot E-ADNI Dataset

**Figure 2:**
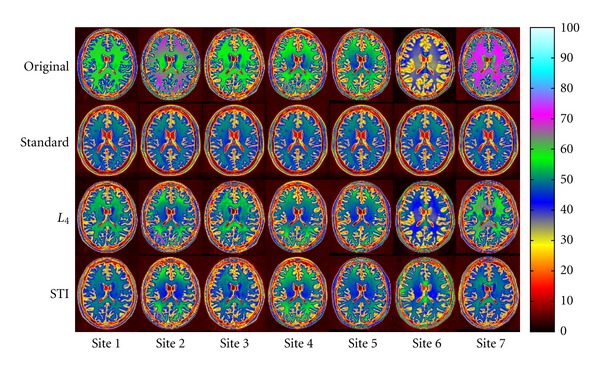
Standardized images for Subject 1 in the Pilot E-ADNI dataset. From top to bottom: linearly registered input images, standard image, and images standardized with *L*_4_ and STI, respectively. From left to right: images for Sites 1 to 7. Color coding enhances intensity differences.

Figure 2 shows standardized *linearly* registered output images for Subject 1 in the Pilot E-ADNI dataset scanned at 7 different sites. We expected standardized intensities to be similar. Qualitatively, STI exhibited a better performance than *L*_4_, especially for WM, and in particular for Sites 6 and 7. We obtained similar results (not shown) for Subjects 2 and 3.

**Figure 3:**
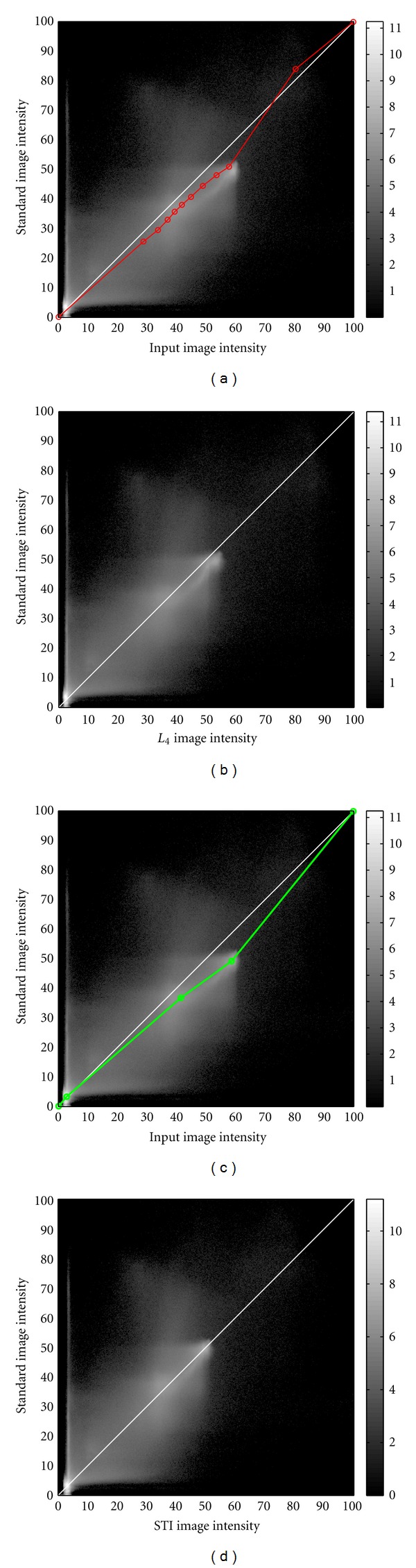
Natural logarithm of the joint intensity histograms obtained for Subject 1 at Site 1, whose images are presented in Figure 2, (a, c) before and (b, d) after standardization with (a, b) *L*_4_ and (c, d) STI. In (a, c), we overlaid the intensity mapping functions obtained with *L*_4_ (a) and STI (c). The histogram diagonals (white) represent perfect intensity correspondence.

Figure 3 presents an example of joint intensity histograms computed before (a, c) and after (b, d) standardization. The grayscale images correspond to the natural logarithm of the joint histograms obtained for Subject 1 at Site 1, whose images are presented in Figure 2. In (a) and (c), we overlaid the intensity mapping functions obtained with *L*_4_ and STI. We observe that STI gives a better intensity correspondence after standardization (d) than *L*_4_ (b).

**Figure 4:**
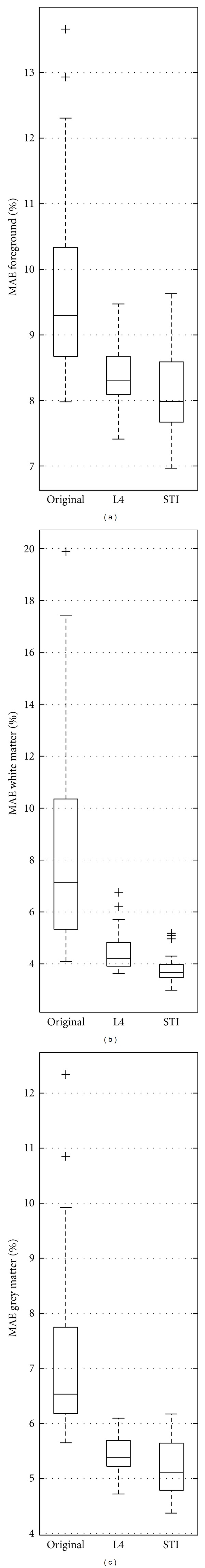
Boxplots of (a) FRG, (b) WM, and (c) GM mean absolute errors for the 21 images from Pilot E-ADNI. (Original) Nonlinearly registered input images, (L4) nonlinearly registered output images standardized with *L*_4_, and (STI) nonlinearly registered output images standardized with STI.

Figure 4 shows MAE boxplots of FRG, WM, and GM over the 21 images. We see that, compared to no standardization (Original), *L*_4_ and STI clearly showed an improvement in terms of MAE for all voxel sets. However, STI offered better performance; the statistical test effectively showed that the difference between *L*_4_ and STI was significant for WM (*P* = 0.0075), almost significant for GM (*P* = 0.0674), but not for FRG (*P* = 0.2459).

### 3.2. ADNI Dataset

**Figure 5:**
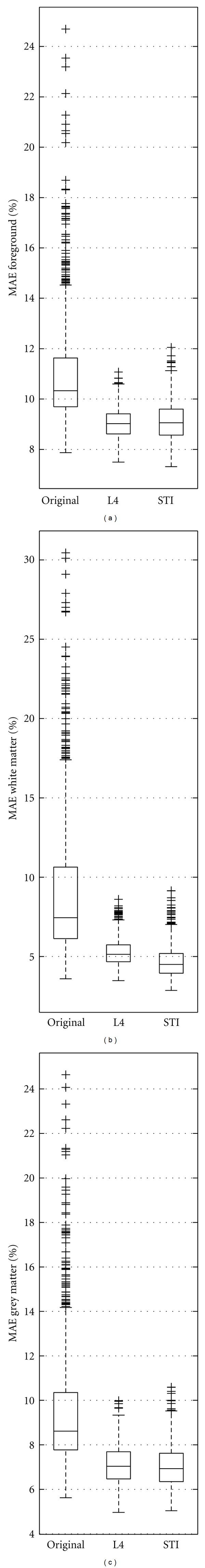
Boxplots of MAE obtained over the 795 images of the ADNI dataset for (a) FRG, (b) WM, and (c) GM. (Original) Nonlinearly registered input images, (L4) nonlinearly registered output images standardized with *L*_4_, (STI) nonlinearly registered output images standardized with STI.

Figure 5 shows MAE boxplots for the 795 different subjects for (a) FRG, (b) WM, and (c) GM. Compared to no standardization (Original), both *L*_4_ and STI exhibited better MAE. STI significantly outperformed *L*_4_ for WM (*P* < 0.0001), with no difference for GM (*P* = 0.3120). However, *L*_4_ was superior for FRG (*P* = 0.0239).

**Figure 6:**
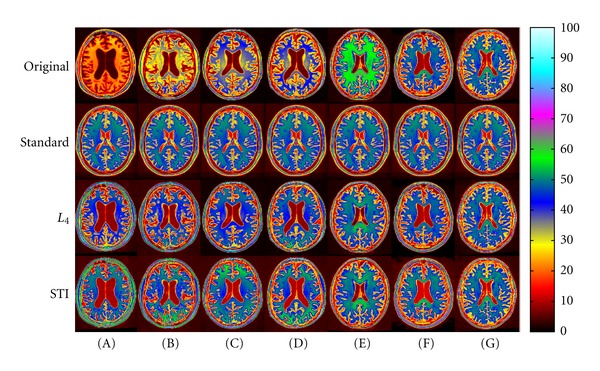
Standardized images of the ADNI dataset, sorted according to MAE percentiles (A) 100, (B) 90, (C) 75, (D) 50, (E) 25, (F) 10, and (G) 0 obtained for the FRG voxel set with *L*_4_. Images (A) and (G) correspond, respectively, to the highest (worst) and lowest (best) MAE obtained in FRG for *L*_4_. From top to bottom: linearly registered input images, standard image, and images standardized with *L*_4_ and STI, respectively. MAE values are given in Table 1.

**Figure 7:**
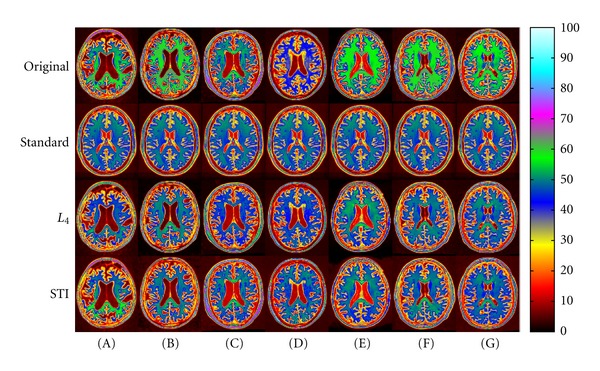
Standardized images of the ADNI dataset, sorted according to MAE percentiles (A) 100, (B) 90, (C) 75, (D) 50, (E) 25, (F) 10, and (G) 0 obtained for the FRG voxel set with STI. Images (A) and (G) correspond, respectively, to the highest (worst) and lowest (best) MAE obtained in FRG for STI. From top to bottom: linearly registered input images, standard image, and images standardized with *L*_4_ and STI, respectively. MAE values are given in Table 1.

Figures 6 and 7 present ADNI image examples sorted according to MAE percentiles 100 (A), 90 (B), 75 (C), 50 (D), 25 (E), 10 (F), and 0 (G) for the FRG voxel set. Images (A) and (G) thus give the highest (worst) and lowest (best) MAE, respectively, for *L*_4_ (Figure 6) and STI (Figure 7). FRG was selected, instead of WM or GM, to avoid any bias toward STI. In fact, selecting FRG would normally favor *L*_4_.

Qualitatively, although FRG MAE decreases from (A) to (G), a corresponding improvement in WM is not necessarily observed. This is also shown in Table 1, where FRG, WM and GM MAE values are given for each image of Figures 6 and 7. MAE values for GM do not necessarily follow the trend for FRG either.

We also see that *L*_4_ and STI can both result in higher (worse) MAE than with no standardization (see Figures 7(A) and 7(C) for WM). In other words, the WM intensity of the nonstandardized image, in these cases, would be closer to the standard than the WM intensity given by *L*_4_ and STI, according to MAE. Over the 795 images of the ADNI dataset, the percentages of images for which *L*_4_ and STI gave higher MAE than without standardization for WM (worst case for both methods) were, respectively, 1.38% and 4.65%. The higher percentage obtained with STI is explained by multiple peaks or wider distributions in the joint intensity histograms of images similar to Figure 7(A). As mentioned, STI selects only the maximum peak for a given tissue. This point is further discussed below.

Finally, for the images presented in Figures 6 and 7, Table 1 reveals that STI gave the lowest MAE values in 26 cases (FRG: 7, WM: 10, GM: 9) versus 16 for *L*_4_ (FRG: 7, WM: 4, GM: 5), even if selecting FRG as the sorting voxel set would have normally favored *L*_4_. It must be noted that this sample is not representative of the whole ADNI dataset, as we artificially selected images to display at each MAE percentiles for each standardization technique. Yet, it is in accordance with boxplots shown in Figure 5 and statistical results detailed earlier.

## 4. Discussion

### 4.1. Methodological Considerations

STI uses spatial correspondence and joint intensity histograms between the input and standard images to find modes and use them as landmarks in the intensity mapping function. While the use of joint histograms has been reported in [13], the authors computed joint histograms between different imaging modalities separately, rather than for the input and standard images. As demonstrated in this study, using such spatial correspondence improves the standardization quality in terms of MAE. This improvement in MAE can impact the final outcome of studies by reducing systematic errors, which in turn can reduce the number of subjects required to achieve a similar level of statistical significance or power.

In this study, the Pilot E-ADNI dataset allowed us to avoid intersubject intensity variations. Effectively, we should theoretically expect that, for a given subject, a similar image be produced for all sites. Compared to *L*_4_, STI was nearer to this expectation, particularly for Sites 6 and 7. Effectively, we showed that STI was significantly superior to *L*_4_ in WM, while differences were not significant in the other voxel sets.

For the ADNI dataset, STI again showed to be significantly better in WM than *L*_4_, while in FRG, *L*_4_ was significantly superior. For FRG, however, we showed that better results did not necessarily correspond to better intensity correspondences for WM and/or GM. This suggests we should not rely on the results obtained in FRG, as long as we are mainly interested in brain GM and WM.

### 4.2. Limitations

We tested STI following linear registration only (results not presented); nonlinear registration yielded better performance. This reliance on registration however remains the main limitation of the technique.

STI is also designed to find one maximum, the mode, in the joint intensity histograms. In cases where two or more peaks are present or the intensity distribution is wider, due to for example insufficient inhomogeneity correction, this might lead to discrepancies such as in Figure 7(A), for which we observe high intensity WM. Although a better preprocessing may solve some of these discrepancies, we plan to add further landmarks in the joint intensity histograms and thus produce better mapping functions in future versions. However, care must be taken to avoid “overstandardizing” intensity variations, especially when dealing with pathologies, for example, severe white matter diseases. We will have to further validate the influence of these pathologies on STI. However, due to the nonlinear registration step, STI should not be sensitive to volume changes, for example, associated to Alzheimer's disease, as suggested by the results obtained with the ADNI dataset.

Another limitation is that STI has been developed for brain T1-weighted MRI. However, it could be easily applied to other sequences (e.g., T2-weighted images), provided a standard image for this acquisition and corresponding tissue masks.

## 5. Conclusion

We presented a new tissue-based standardization technique called STI. This technique uses spatial correspondence between an input image and a standard determined via global linear and nonlinear registration. Registration allows the use of joint histograms to determine intensity correspondence in each tissue, defined within voxel masks.

We compared STI to an existing histogram-matching technique and showed that STI was superior in terms of mean absolute error, particularly in the white matter, in two multicentric datasets. These results demonstrated that standardization techniques should not be aimed solely at matching histograms and that spatial information should also be incorporated. To our knowledge, it is the largest study on intensity standardization.

## Figures and Tables

**Table 1:** MAE (%) of the ADNI images presented in Figures 6 and 7, obtained for FRG, WM, and GM.

Figure	Voxel set	Technique	(A)	(B)	(C)	(D)	(E)	(F)	(G)
Figure 6	FRG	Original	21.27	13.93	11.94	12.14	9.90	8.85	7.87
		*L*_4_	11.07	9.84	9.41	9.02	**8.62**	**8.30**	**7.50**
		STI	**9.93**	**9.71**	**9.24**	**8.81**	8.69	8.48	7.56
	WM	Original	27.89	16.65	12.90	11.28	6.88	5.08	4.04
		*L*_4_	8.60	7.05	6.32	4.59	**4.19**	5.04	3.67
		STI	**4.42**	**4.86**	**4.03**	**4.04**	4.40	**4.34**	**3.64**
	GM	Original	22.61	14.31	11.24	10.77	7.58	7.20	5.62
		*L*_4_	9.99	8.58	7.52	6.65	**6.04**	6.70	**4.97**
		STI	**8.75**	**8.05**	**7.03**	**6.50**	6.43	**6.50**	5.31

Figure 7	FRG	Original	12.14	11.77	10.10	10.90	10.13	10.29	8.93
		*L*_4_	**10.64**	**9.70**	**8.88**	**8.92**	8.77	8.24	7.67
		STI	12.05	10.11	9.60	9.05	**8.57**	**8.23**	**7.32**
	WM	Original	8.04	9.38	5.34	10.01	7.68	8.30	6.43
		*L*_4_	**6.49**	**5.26**	6.32	5.70	4.95	**4.28**	4.02
		STI	8.24	5.94	**5.00**	**4.11**	**4.89**	4.32	**3.69**
	GM	Original	10.72	10.00	8.30	9.21	8.59	8.19	7.37
		*L*_4_	**8.99**	**7.63**	7.71	6.83	6.90	**6.23**	5.89
		STI	10.59	8.12	**7.69**	**6.70**	**6.79**	6.29	**5.49**

Best (lowest) MAE values are highlighted in bold characters.

## Abbreviations

2D: Two-dimensional
ADNI: Alzheimer's Disease Neuroimaging Initiative
BKG: Background
CSF: Cerebrospinal fluid
E-ADNI: European ADNI
FRG: Foreground
GM: Grey matter
MAE: Mean absolute error
MRI: Magnetic resonance imaging
STI: *STandardization of Intensities*
WM: White matter.
